# Augmenting Deep Learning Performance in an Evidential Multiple Classifier System

**DOI:** 10.3390/s19214664

**Published:** 2019-10-27

**Authors:** Jennifer Vandoni, Sylvie Le Hégarat-Mascle, Emanuel Aldea

**Affiliations:** 1 SATIE-CNRS UMR 8029, Paris-Sud University, Paris-Saclay University, 91405 Orsay CEDEX, France; jennifer.vandoni@gmail.com (J.V.); emanuel.aldea@u-psud.fr (E.A.); 2 SAFRAN SA, Safran Tech, Pole Technologie du Signal et de l’Information, 78772 Magny-les-Hameaux, France

**Keywords:** deep learning, ensemble classifiers, Belief Function Theory, pedestrian detection, high-density crowds

## Abstract

The main objective of this work is to study the applicability of ensemble methods in the context of deep learning with limited amounts of labeled data. We exploit an ensemble of neural networks derived using Monte Carlo dropout, along with an ensemble of SVM classifiers which owes its effectiveness to the hand-crafted features used as inputs and to an active learning procedure. In order to leverage each classifier’s respective strengths, we combine them in an evidential framework, which models specifically their imprecision and uncertainty. The application we consider in order to illustrate the interest of our Multiple Classifier System is pedestrian detection in high-density crowds, which is ideally suited for its difficulty, cost of labeling and intrinsic imprecision of annotation data. We show that the fusion resulting from the effective modeling of uncertainty allows for performance improvement, and at the same time, for a deeper interpretation of the result in terms of commitment of the decision.

## 1. Introduction

Even though deep learning solutions tend to outperform the other supervised learning techniques when trained on large amounts of data, applying them effectively in presence of few labeled data is nowadays an open issue. Most of the existing works are devoted to finding the best network for applications for which huge datasets exist, but few attention is given to specific real-setting problems where training data are hard to obtain and therefore out-of-the-box networks may be impossible to be trained. Nonetheless, in recent years, many regularization techniques have been proposed to tackle the problem of overfitting, from data augmentation to early stopping and dropout, besides the traditional weight decay. These techniques used together could help in applying deep learning techniques in the presence of small datasets. In addition to these techniques, fusion with another strong classifier may be considered.

Simultaneously, a criticism that is often made of deep learning methods is the fact that they act like “black-boxes”, making it hard for their users to interpret the obtained results. This limitation is highly relevant when learning from small amounts of data, where a measure of model uncertainty would be particularly important. To this extent Bayesian Neural Networks (BNNs, Bayesian NNs) offer a probabilistic interpretation of deep learning models by inferring distributions over the models’ weights, allowing to measure model uncertainty, but they are usually practically limited. Recently, an ensemble-based method relying on the use of dropout at inference time has been proposed in [1] (Monte Carlo dropout), allowing to obtain several realizations sampled from the same network with randomly dropped-out units at test time, from which a confidence measure on the prediction can be derived.

Following this line of work, we intend to investigate the use of deep learning techniques in presence of small training datasets for specific applications (in our case high-density crowd pedestrian detection). A solution proposed for instance by [2] in the case of hyperspectral data is to reduce the number of weight parameters required to train the model by considering some constraints related to the physical interpretation of the weights. In this work, the type of the data (grayscale images) is not suitable for such prior constraints, we propose the use of an ensemble method that is justified according to two different reasons. Firstly, it acts as another regularization technique to mitigate the risk of overfitting; secondly, it allows us to measure the model confidence about each prediction. To this extent, we propose to work in the context of the Belief Function Theory [3,4,5] (BFT) to better leverage the classifier’s unique properties. The evidential framework [6,7,8,9,10] is indeed able to naturally model the concept of *imprecision* in addition to the uncertainty value provided by the classifiers.

We thus propose an evidential Multiple Classifier System (MCS), which is in turn composed by two ensembles of classifiers. The first one, called CNN-ensemble, is an ensemble of convolutional neural networks (CNNs) derived using the Monte Carlo dropout technique. The second one, called SVM-ensemble, is an ensemble of Support Vector Machine (SVM) classifiers trained with different descriptors in an active learning (AL) Query-by-Committee fashion previously proposed in [11]. Specifically, starting from a single sensor input, i.e., an image lattice, we derive two different ensembles based on complementary classifiers. These two ensembles are then considered as different information sources, like virtual sensors.

We apply the proposed Evidential MCS to the difficult application of high-density crowd pedestrian detection for multiple reasons. Indeed, although in the last years, many efforts have been devoted to improve the performance of pedestrian detection [12], baseline methods cannot be always applied in crowded contexts because of scarce labeled data, and intrinsic differences with respect to the sparse case which may be cause of imprecision in the final detection results.

Pedestrian detection by itself is noticeably one of the most challenging categories of object detection. There exists indeed a large variability in the local and global pedestrians’ appearance, due to the variety of possible body shapes, or different styles and types of clothes and accessories which may alter the silhouettes of the individuals. Besides, in real-world scenarios several people can occupy the same region, partially occluding each other, and this phenomenon becomes more prevalent as the crowd density increases.

Traditionally, in the context of supervised learning for pedestrian detection, the Histogram of Oriented Gradients (HOG) descriptor [13] has been proposed for the scope, but its performance can be easily affected by the presence of background clutter and occlusions. Alternatively, deformable part-based models [14] consider the appearance of each part of the body and the deformation among parts for detection. In parallel with the development of traditional approaches, more sophisticated methods [15] proposed a cascade Random Forest classifier with a census transform histogram visual descriptor. Recently, neural networks have been employed in the context of pedestrian detection, either in conjunction with hand-crafted features [16,17], or in a stand-alone manner [18,19,20,21]. In [22], deep features, deformation handling, occlusion handling, and classification are jointly learnt for pedestrian detection. Late fusion of multiple convolutional layers has been recently proposed in [23] relying on Region Proposal Networks (RPNs), showing that earlier convolutional layers are better at handling small-scale and partially occluded pedestrians. A Scale-Aware Fast R-CNN framework has been also proposed in [20]. Again, in presence of dense crowds, the region proposal step loses its interest as the number of targets becomes too large to be tractable. Finally, let us recall that if large datasets with dotted annotations corresponding to head’s center coordinates are available for applications such as cross-scene people counting, only little attention has been given to specific scenes for the more difficult task of pedestrian detection. For all the highlighted limitations, a straightforward extension of the techniques designed for pedestrian detection in non-crowded scenes is not suitable for dealing with crowded situations.

In this work, we show that deep learning techniques can still be used in conjunction with a traditional classifier even in such difficult situations, where we are interested in specific scene analysis for which labeled data is scarce and not precise (i.e., we just have the head’s center coordinates instead of bounding boxes or precise segmentation maps, contrarily to traditional pedestrian detection setting). This paper is organized as follows. Section 2 describes the proposed methodology that allows for combination of CNN-ensemble and SVM-ensemble. Firstly, we propose a fully convolutional network especially designed to recover small objects and we create a CNN-ensemble through Monte Carlo dropout. The whole output is interpreted in the Belief Function framework. Then, having described the SVM-ensemble derived from [11] work, we explain how the two ensembles are fused together in the context of BFT. Section 3 shows to which extent the proposed approach has been able to improve the overall detection results even in such a difficult setting, by analysing the obtained results. In this way, we prove that deep learning techniques can be applied also in presence of extremely small datasets for solving targeted problems, and can benefit from the fusion with another strong classifier. Finally, Section 4 draws the conclusions of this study.

## 2. Evidential Multiple Classifier System

In this section we go through the details of the proposed evidential MCS for pedestrian detection. This method is particularly useful in presence of scarce training data, where recent deep learning techniques may fail to obtain reliable predictions. The simultaneous use of two different ensemble of classifiers, namely a deep learning-based one and a SVM-based one, allows us to exploit their different strengths in order to obtain a robust prediction. Note that this is not straightforward, since in presence of few, strong classifiers the fusion strategy must be particularly well-designed in order to exploit their respective peculiarities, and for this reason we propose a fusion in the context of BFT which allows us to obtain a measure of localized imprecision in addition to more robust predictions.

**Figure 1:**
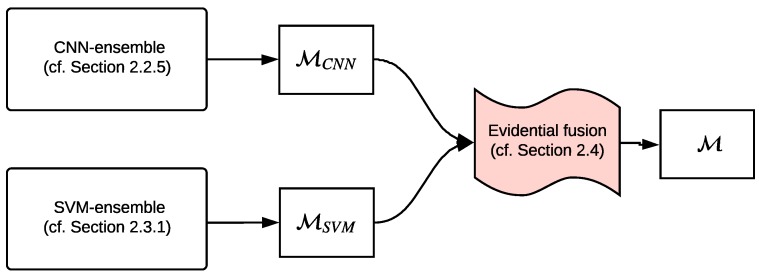
Proposed evidential MCS scheme.

Figure 1 shows a simplified scheme of our approach which combines two ensembles of different classifiers in an evidential framework. The CNN-ensemble is obtained by sampling the posterior distribution of the proposed FE + LFE network through Monte Carlo dropout technique [1], while the SVM-ensemble is obtained by exploiting different hand-crafted features in an active learning framework. The output of those ensembles, $M_{CNN}$ and $M_{SVM}$ respectively, are then combined in the context of BFT to obtain the final output map M. In the following, after a brief introduction of the BFT that will play a key role in our method to model the concept of imprecision, we firstly detail the proposed CNN-ensemble, then we recall the SVM-ensemble, already proposed in [11], and finally, we explain their fusion procedure in the context of BFT.

### 2.1. Modeling Imprecision with BFT

To handle both the uncertainty and the related imprecision that, in our case, may come from the specific classifier and/or data used in the training process, BFT [3,4] is designed to handle compound hypotheses. If Θ denotes the set of mutually exclusive hypotheses (i.e., the discernment frame), belief functions are defined on the powerset $2^{\Theta }$. In our case, denoting by *H* and $\bar{H}$ the two singleton hypotheses, *“Head”* and *“Not Head”*, the discernment frame is $\Theta =\left\{H,\bar{H}\right\}$, and the set of hypotheses is $2^{\Theta }=\left\{Ø,H,\bar{H},\left\{H,\bar{H}\right\}\right\}$.

The *mass* function noted *m* is the *Basic Belief Assignment* (BBA) that satisfies $\forall A\in 2^{\Theta },m\left(A\right)\in \left[0,1\right]$, $\sum_{A\in 2^{\Theta }}m\left(A\right)=1$. The hypotheses for which the mass function is non-null are called *focal elements*. If only singleton hypotheses are focal elements, the BBA is called Bayesian.

Then, other BF are in one-to-one relationship with *m*. In this particular setting in which we have only two singleton hypotheses and considering m(Ø)=0, the *plausibility* and the *credibility* functions noted Pl and Bel respectively are defined by: $\forall A\in \left\{H,\bar{H}\right\},Bel\left(A\right)=m\left(A\right)\;\mathrm{and}\;Pl\left(A\right)=m\left(A\right)+m\left(\Theta \right)$. Pl and Bel are dual functions: $\forall A\in 2^{\Theta },Pl\left(A\right)=1-Bel\left(\bar{A}\right)$ (where $\bar{A}$ denotes the complement of *A* with respect to Θ). Since in our case we work with only two singleton hypotheses, the equations simplify and we will introduce them directly when needed. However, we encourage the interested reader to refer to the seminal works [3,4] for a more detailed explanation about BFT foundations.

### 2.2. CNN-Ensemble

#### 2.2.1. Representing Model Uncertainty in Deep Learning

Obtaining a measure of uncertainty of a model trained with deep learning techniques is not trivial. The general training of deep learning models allows us to obtain the best model parameters through backpropagation, but they are usually only point estimates. These parameters are then kept fixed at inference time in the forward pass to perform prediction. However we cannot easily know whether a trained model is certain about its output, and no classifier is able to directly provide credible or confidence intervals about its predictions.

We want the network to be able to measure predictive uncertainty, that is the confidence it has with respect to the prediction it makesThis is particularly important for applications related to real-settings such as autonomous driving or security access to critical systems, where relying on model uncertainty to adapt decision making is crucial, and generally for applications for which only a small amount of data is available for the training.

To tackle this issue, BNNs have been firstly studied extensively in [24,25] and more recently in [26,27,28], sometimes being referred to as variational techniques. They are based on the observation that an infinitely wide neural network with distributions placed over its weights converges to a Gaussian process [25], thus, considering finite neural networks (NNs), they are mathematically equivalent to an approximation of the probabilistic deep Gaussian process [29]. In order to obtain model uncertainty estimates, BNNs place a prior probability distribution over each networks’ weight. In this way, they potentially offer robustness to overfitting during training along with uncertainty estimates about the predictions. However, the applicability of these types of models is quite limited, and they have not been largely followed up by the deep learning community. If on the one hand Bayesian probability theory offers mathematically grounded tools to reason about model uncertainty, on the other hand they come usually with prohibitive computational costs. BNNs have shown indeed to be quite difficult to work with, often requiring the optimization of many more parameters with respect to standard networks.

Recently, the authors of [1] developed a new theoretical framework by casting *dropout* (and its variants) in deep NNs as approximate Bayesian inference in deep Gaussian processes. The foundation of this theory directly provides tools to model the uncertainty without the need to change neither the model architecture nor the objective function. The authors have shown that a neural network with arbitrary depth and non-linearities, with dropout applied at every layer, is mathematically equivalent to an approximation of the probabilistic deep Gaussian process. This means that the optimal weights found through the optimization of a NN with dropout are the same as the optimal variational parameters in a Bayesian NN with the same structure. Further, this means that a network already trained with dropout *is* indeed a BNN. Moreover, this result is valid not only using dropout, but also its variants such as DropConnect [30] or multiplicative Gaussian noise [31], i.e., using any Stochastic Regularization Technique (SRT). SRTs are techniques used to regularize a deep learning model through the injection of stochastic noise directly into it (the most popular technique is dropout which switches off units with respect to a previously set probability). The intuition is that SRTs approximately integrate over the models’ weights, so that they can be interpreted as performing approximate inference and, as a result, uncertainty information can be extracted.

Practically, after training the network, Monte Carlo (MC) methods are used at test time to draw samples from a Bernoulli distribution across the network’s weights, by performing *T* stochastic forward passes through the network with dropout. This is why the method is known as *MC-dropout*. Note that this does not require any additional parametrization, and from this it is easy to derive the sample mean by averaging the results and the standard deviation that can be interpreted as predictive uncertainty. In this way, we can obtain an ensemble of classifiers composed by *T* different realizations given by dropping out different units of the network at each forward pass. This method has several advantages, i.e., it is easily adaptable to complex models and does not require any change to the model architecture or optimization procedure (the training is only performed once, while dropout is applied at test time), besides being very easy to implement in practice. MC-dropout has been successfully applied in different applications, from segmentation for scene understanding [32] to camera re-localization [33], allowing to model the predictive uncertainty through standard deviation of the stochastic realizations obtained with dropout.

We shall finally mention a different but related approach, that cannot be considered as approximate inference in BNNs, but which may nevertheless be used to estimate model uncertainty relying on ensemble learning. This technique builds an ensemble of deterministic models (each model in the ensemble produces a point estimate rather than a distribution) by independently training the same network on the same dataset many times with different weight initialization. Then, at inference time, an average is done in order to get a prediction, while the predictive uncertainty is measured through the variance of the outputs of all the models. Very recently, reference [34] proposed *deep ensembles* based on this idea, relying also on adversarial training [35,36] to smooth predictive distributions, treating the ensemble built in this way as a uniformly-weighted mixture model and approximating the ensemble prediction as a Gaussian whose mean and variance are the ones of the mixture respectively. However, this approach is not always suitable for a number of reasons. Firstly, training many neural networks can be a long process. Secondly, even if this approach is anyhow computationally more efficient than many Bayesian approaches presented in the previous section, its produced uncertainty estimates lack in many ways [37].

Now, we firstly detail the ground truth construction, then we describe the architecture we designed for our application, namely pedestrian detection in high density crowds, and then we formulate its Bayesian counterpart that makes use of MC-dropout to get samples from the posterior distribution over the network’s weights. Finally, we move to the BFT to explain the proposed BBA allocation and combination.

#### 2.2.2. Soft-Labeling

In our specific environment, precise labeling to perform head detection is usually impossible to achieve, due to the presence of clutter and occlusion problems that make the contour of the heads barely distinguishable from the background, in addition to the very small size of the targets. A precise definition of head borders is thus difficult even for a human operator. Therefore, commonly used datasets do not come with precise segmentation ground-truth but rather with just a list of coordinates that indicate the center of the heads. For these reasons, we investigate the problem of head detection from partially labeled data, namely where only the center of each head is dot-annotated, with only a prior knowledge about the average radius of a head in pixels (possibly with respect to its location in the image in case of strong perspective variation due to camera tilt). Note that this is a specific setting of more general case of imprecise objects definition, for which possible different imprecise shapes could be considered.

Starting from the dotted annotation, instead of simply performing a dilation with a circular structuring element centered in each annotation location, that would result in a binary map with possibly incorrect labels assigned to pixels corresponding to head’s boundaries, we propose a *soft label* definition of the ground-truth map, as often done in the context of crowd density estimation [38,39,40]. Starting from the binary ground-truth map with 1-valued label for each head center location $\left(x_{c},y_{c}\right)$, we apply a cumulative Gaussian smoothing such that the ground-truth map for each head is expressed in terms of a Gaussian distribution as:

(1) $$\left(x,y\right)∼\eta \cdot \sum_{c\in C}N\left(\left(x_{c},y_{c}\right),\sigma_{h}\right),$$

where η is a scaling factor to face the class imbalance problem, while $2\sigma_{h}$ is the expected head radius and C is the set of dots annotating ground-truth heads.

**Figure 2:**
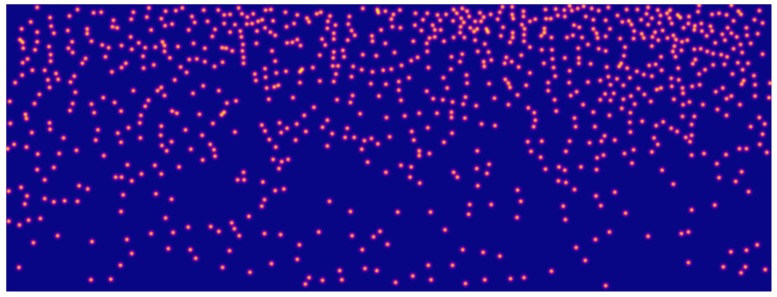
Typical example of a ground-truth map as cumulative Gaussian distributions, one per head. The score associated to each pixel of the ground-truth map is the sum of the contributions of each Gaussian at the given location. In the image, scores span from blue (low) to yellow (high).

We consider Gaussian distributions as they are infinitely differentiable functions presenting tails which vanish at infinity, being able to model well the imprecision on the head contour locations. We apply a cumulative Gaussian smoothing in the sense that the final ground-truth map is the sum of Gaussian distributions derived from each head center locations. The resulting map is not a probability distribution by itself, but rather the score associated to each pixel represents the sum of probabilities that any head, occluded or not, is located at that position, directly facing in this way also the problems of close and occluded heads. In presence of close heads indeed, maxima would still indicate the head center locations, while in presence of occluded heads the evidence of the partially visible head will be reinforced through the cumulative sum. Ground-truth Gaussian smoothing are finally also able to mitigate location errors in the annotated ground-truth, that could have a higher impact considering sharp-defined objects in presence of small targets. Figure 2 shows a typical example of a ground-truth map obtained with the proposed soft labels on an example image from the Makkah dataset that will be later employed to show experimental results.

#### 2.2.3. FE + LFE Network

We choose to cast the specific problem of head detection in high-density crowds as a segmentation task, in the sense that we want to assign a different label to each different pixel of the image, depending whether or not it belongs to a head. Given an input image, we aim thus at performing *dense* prediction by estimating an output map of the same size of the input. However, attention must be payed with respect to two different concerns related to our application, namely the impossibility to obtain a precise ground-truth map and the impossibility to have huge labeled datasets at our disposal. These two aspects will be investigated in the following, as they are both possible causes of imprecision that can be modeled through the BFT.

Among the various architecture for semantic segmentation, we propose a network inspired by [41] that makes use of dilated convolution to be able to recover small objects, proposed in the field of remote sensing imagery. Note that we tried to use also UResNet [42], an encoder-decoder network inspired by both U-Net [43] and ResNet using residual blocks, but the training data at our disposal resulted to be not enough to train this type of network with a too high number of parameters.

In the context of remote sensing image analysis, the authors of [41] highlighted a major problem of segmentation in presence of small and densely aggregated objects. The use of pooling layers indeed tends to degrade the output resolution so that details of the very small objects are lost. In these situations, even the use of shortcuts like skip-connections in the U-Net could not be enough to recover small targets. Pooling layers are however important, for two different reasons. Reducing the spatial dimension of the 3D volume, they allow for a larger context consideration without increasing the receptive field of the filters, and to reduce the number of total parameters to be learned by the network.

In order to enlarge the receptive field of the filters going deeper with the layers, without degrading the output resolution nor increasing the filters’ size (thus increasing the number of parameters), dilated convolutions can be exploited. Linearly increasing the dilation factor through the layers’ chain will result in an exponential enlargement of the receptive field that is therefore able to capture larger context and recover smaller objects. Context information is indeed crucial in recovering small objects, as pointed out in [44].

The authors of [41] however noticed that aggressively increasing dilation factors through the network’s layers in a straightforward way is detrimental in aggregating local features. Dilation causes weights to skip information between cells, and this results in a bad modelisation of the structure of small objects, presenting grid patterns in the final output. To solve this problem, they propose a network without pooling layers that conversely concatenates a Front End (FE) module of increasing dilation factors, with a Local Feature Extraction (LFE) module of decreasing dilation factors, arranged in a symmetrical way. The FE module is thus able to consider larger context for small objects detection, while the LFE module enforces the spatial consistency of the output by gathering spatial information decreasing the dilation size.

The Front End architecture employed in [41] is a VGG network [45] deprived of the final classifier layer (to obtain a fully convolutional network) and deprived of the max pooling layers. Instead of the latter, dilation factors are increased at the corresponding network depth. Then, the LFE module keeps invariant the number of filters and the kernel size, while decreasing the dilation factors up to one, in a specular way with respect to the FE.

The only drawback of such an architecture is that with respect to the U-Net it needs more memory to perform backward and forward passes, because feature maps at each layer have always the same size as the original input since there is no pooling operation. Thus, we propose a modified architecture (shown in Table 1) that keeps the memory use manageable by reducing the number of filters at each convolutional layer. The choice of the reduction of the number of filters per layer has nevertheless two purposes. On the one hand, it allows us to fulfill hardware constraints, while on the other hand it helps in preventing the network to overfit, as we know we are going to train it with small datasets. To this extent, also batch normalization has been added on top of each convolutional layer, for faster convergence [46] by reducing internal covariate shift.

Note that to obtain a “simpler” network to prevent overfitting we could have conversely decreased the number of total layers. However, we preferred to reduce the number of filters per layer for two reasons. Firstly, decreasing the number of layers would have prevented the network to learn more complex features. Secondly, we would have not entirely exploited the benefit of increasing/decreasing dilation factors. Moreover, we found that adding many layers with a dilation factor of 3 was too heavy, and for this reason we preferred to add a single central layer with such a big dilation (however, this depends on the expected size of the targets).

Another modification to the usual structure of fully convolutional networks regards the last layer. Usually, the layers’ chain terminates with a convolutional layer, without any activation function that would apply a non-linear transformation which is usually not needed. In our specific application however, the desired output values are real values which are possibly positively unbounded (a pixel’s score is indeed the sum of contributions given by all the surrounding Gaussian distributions representing surrounding heads), while at the same time loosing their meaning as long as they take values under zero. By definition, being a cumulative sum of Gaussian distributions, the ground-truth maps obtained through the soft labeling procedure contain values which are always greater than or equal to zero. In this way, by simply integrating the map over a region of interest we can obtain the number of people present in that area. By setting the last layer as a convolutional one however, we would allow the network to possibly produce negative output values which would be the origin of noise in the estimation of the number of people, complementary application to pedestrian detection in the context of crowd macroscopic analysis [40].

For this reason, we propose to use an activation function in the last layer which bounds the values at zero, like the sigmoid or the ReLU. For example a sigmoid activation as last layer is used in [47], to constrain the output values between 0 and 1. For our particular application however, the sigmoid is not suited for two main different reasons. Firstly, since the sigmoid function tends toward zero without really reaching it, punctual noise would become more evident. Secondly, since it saturates at 1, we loose the meaning of “cumulative” output: when two heads are one next to the other, they would result in a single large blob and the score of each pixel would no more represent the cumulative sum of surrounding heads contributions.

On the contrary, we propose to use a ReLU activation function in the last layer. It has the effect of a threshold, setting all the negative values to zero. Nevertheless, since it is integrated inside the network, it has beneficial effects on backpropagation convergence with respect to a simple post-processing thresholding. In this way, the network learns easily to return zero for background pixels, being able at the same time to suppress a part of the background noise. The local density estimation is therefore also enhanced, since the network looses its tendency to compensate between low and high values adding noise, as highlighted in [40].

#### 2.2.4. Bayesian FE + LFE Network

Now, we want to formulate the Bayesian counterpart of the proposed FE + LFE network that uses MC-dropout to get samples from the posterior distribution over the network’s weights. Note that the “Bayesian” prefix refers here to the Bayesian probability theory, and not to the Belief Function framework meaning which simply stands for null-mass BBAs except for singleton hypotheses.

For the definition of the Bayesian FE + LFE we follow the formulation of Bayesian SegNet [32], which is built upon SegNet [48] network for pixel-wise segmentation.

Given a list of training inputs x and corresponding outputs *y*, we are interested in finding the posterior distribution over the network’s convolutional weights, W:

(2) p(W|x,y),

However, this posterior distribution is generally intractable, and needs to be approximated [49]. To this extent, variational inference can be used, that allows us to approximate the intractable posterior through a function q(W) over the network’s weights. This function is learned by minimizing the Kullback-Leibler (*KL*) divergence between this approximating distribution and the actual posterior:

(3) $$D_{KL}\left(q\left(W\right)||p\left(W|x,y\right)\right).$$

As proposed in [50], given a CNN with *L* layers of dimension K×K, we define the approximating variational distribution $q(W_{i})$ for every convolutional layer *i* with units *j* as:

(4) $$\begin{array}{ccccc} W_{i} & = & M_{i}\cdot \mathrm{diag}\left({\left[z_{i,j}\right]}_{j=1}^{K_{i}}\right), & & \\ z_{i,j} & ∼ & \mathrm{Bernoulli}\left(p_{i}^{\mathrm{drop}}\right) & i=1,\ldots ,L, & j=1,\ldots ,K_{i-1}, \end{array}$$

where $z_{i,j}$ are Bernoulli distributed random variables with probabilities $p_{i}^{\mathrm{drop}}$ (i.e., dropout probabilities), and $M_{i}$ contains the variational parameters to optimize. Note that dropout probabilities $p_{i}^{\mathrm{drop}}$ could also be optimized, but as in [32] we kept them fixed to an equal constant value found through validation. In this way, as proven in [50], we obtain the approximate model of the Gaussian process.

Now, we train the network and we sample the posterior distribution over the weights using dropout at test time, performing *T* different forward passes through the network. As a result for a given testing image we obtain *T* different realization maps ${\hat{M}}_{1},\ldots ,{\hat{M}}_{T}$, output of different dropout-perturbed versions of the original network. Classically, the mean map $M_{\mu }$, given by the mean value evaluated independently for each pixel, is interpreted as the final prediction map, while the standard deviation map $M_{\sigma }$ is interpreted as an estimate of the predictive uncertainty. However, we propose once again to work in the BF framework, that we consider more suited to model the specific imprecision of each different realization obtained with dropout. In the following, we will explain the proposed BBA allocation for every realization that will allow us to perform a robust fusion among them as well as to obtain evidential measures of predictive uncertainty for every pixel of the final output map.

#### 2.2.5. CNN-Ensemble BBA Allocation and Combination

While being an easy yet mathematically grounded approach to obtain a measure of uncertainty out of any kind of deep network, MC-dropout has some drawbacks. Firstly, for practical reasons, often we can perform only a limited number of forward passes with dropout, and the mean value could not robust in presence of outliers. Secondly, as reported in [37], the obtained uncertainty is not calibrated (it can scale differently for different datasets) and usually underestimated (variational inference is known indeed to underestimate predictive variance).

Leaving partially apart the mathematical ground in favor of an analysis more adapted to our specific setting, we note that the median is a more robust estimator than the average in presence of outliers. In the same way, instead of relying on the standard deviation, we can better employ the Median Absolute Deviation (*MAD*) [51], which is a robust measure of the variability of a univariate sample of quantitative data, more resilient to outliers than the standard deviation.

In our context, given the *T* realization maps, the *MAD* map $M_{MAD}$ is defined as:

(5) $$M_{MAD}=\mathrm{median}\left(\;|{\hat{M}}_{i}-\mathrm{median}\left({\left\{\hat{M}\right\}}_{1}^{T}\right)|\;\;\right),$$

where $\mathrm{median}\left({\left\{\hat{M}\right\}}_{1}^{T}\right)$ is the median over all the *T* realizations.

Finally, we propose to rely on the Belief Function framework to obtain a better estimation of the model imprecision given the *T* realizations that can be interpreted as different sources for an evidential combination. BBA allocation is then performed as follows.

We have *T* maps ${\hat{M}}_{1},\ldots ,{\hat{M}}_{T}$ which correspond to the *T* output realizations obtained with forward passes through the network with dropout, and we are interested in finding a BBA allocation to perform the combination among them and derive evidential measures of imprecision.

Firstly, we can derive Bayesian BBA maps $M_{1}^{B},\ldots ,M_{T}^{B}$, where a BBA is associated to each pixel x of each realization, so that we obtain *T* maps of BBAs $\left\{m_{x,t}^{B},x\in P\right\}$, where P is the pixel domain and t=1,…,T. These Bayesian BBAs maps are 4-layers images where each layer corresponds to the mass values of any hypothesis in $\left\{Ø,H,\bar{H},\Theta \right\}$. $M_{t}^{B}\left(A\right)$ corresponds to the layer image associated to hypothesis *A* for the realization (source) *t*. Note that in this preliminary Bayesian BBA allocation, layer images corresponding to non-singleton hypotheses are null, by definition. So, for each source *t*, with t=1,…,T:

(6) $$\begin{array}{c} \left\{\begin{array}{ccc} M_{t}^{B}\left(Ø\right) & = & {\left\{0\right\}}_{x\in P},\\ M_{t}^{B}\left(H\right) & = & {\hat{M}}_{t},\\ M_{t}^{B}\left(\bar{H}\right) & = & 1-{\hat{M}}_{t},\\ M_{t}^{B}\left(\Theta \right) & = & {\left\{0\right\}}_{x\in P}. \end{array}\right. \end{array}$$

Now, we want to take into account the reliability of the pixel-wise prediction given by every source in order to perform a pixel-wise tailored discounting. Note that this would be impossible in the probabilistic framework; moreover, we are not just computing an overall source discounting, but rather each pixel of each source will be discounted differently on the basis of its reliability.

To measure this latter, we take inspiration from the *MAD*. For each source *t*, we compute a discounting coefficient map $\Gamma_{t}\;:\;{\left\{\gamma_{x,t}\right\}}_{x\in P}$ such that a different coefficient $\gamma_{x,t}$ is associated to every pixel of each source,

(7) $$\Gamma_{t}=\alpha \left(1-\left(\left|{\hat{M}}_{t}-\mathrm{median}\left({\left\{\hat{M}\right\}}_{1}^{T}\right)\right|\right)\right).$$

In this way, we discount more the pixels whose value is more distant to the median value among the *T* realizations, since they are supposed to be less representative (even possibly outliers). The α parameter is a scaling factor which allows us to control the amount of discounting.

Applying the proposed discounting, we derive the following BBA maps for every source *t*: $\forall A\in \left\{H,\bar{H}\right\}$,

(8) $$\begin{array}{c} \left\{\begin{array}{ccc} M_{t}\left(Ø\right) & = & {\left\{0\right\}}_{x\in P},\\ M_{t}\left(A\right) & = & \Gamma_{t}\;⋆\;M_{t}^{B}\left(A\right),\\ M_{t}\left(\Theta \right) & = & {\left\{1\right\}}_{x\in P}-M_{t}\left(H\right)-M_{t}\left(\bar{H}\right), \end{array}\right. \end{array}$$

where $M_{1}⋆M_{2}$ represents the Hadamard product between matrices $M_{1}$ and $M_{2}$.

To combine the *T* different maps to obtain a single output map M with BBAs associated to each pixel x, i.e., ${\left\{m_{x}\right\}}_{x\in P}$, we use the conjunctive combination rule [3]. Note that using this rule instead of a cautious rule (e.g., [52]) devoted to non-independent sources, we consider that the different dropout realizations correspond to cognitively independent sources even if sampled from the same distribution.

In our case where $\left|\Theta \right|=2$, the analytic result using the conjunctive combination rule may be easily derived: $\forall A\in \left\{H,\bar{H}\right\}$,

(9) $$\begin{array}{c} \left\{\begin{array}{ccc} m_{x}\left(A\right) & = & \sum_{\begin{array}{c} \left(B_{1},\ldots ,B_{T}\right)\in {\left\{A,\Theta \right\}}^{T},\\ \exists t\in \left[1,T\right]s.t.B_{t}=A \end{array}}\prod_{t=1}^{T}m_{x,t}\left(B_{t}\right),\\ m_{x}\left(\Theta \right) & = & \prod_{t=1}^{T}m_{x,t}\left(\Theta \right),\\ m_{x}\left(Ø\right) & = & 1-m_{x}\left(H\right)-m_{x}\left(\bar{H}\right)-m_{x}\left(\Theta \right). \end{array}\right. \end{array}$$

The result is thus a four-layer map $M_{CNN}$ of BBAs $m_{x}$, that can be used to derive evidential measures of uncertainty about the network prediction. To this extent, we can obtain the ignorance map as $M{\left(\Theta \right)}_{CNN}$, that represents the remaining ignorance which has been decreased by the combination but not completely solved, indicating a lack of sufficient information during training to perform a reliable prediction. Likewise, $M{\left(Ø\right)}_{CNN}$ is often interpreted as a conflict map [53], and presents higher values for pixels whose prediction completely disagrees through the various realizations.

Finally, in every pixel x the decision is taken from $m_{x}$. Pignistic probability may be used to give a probabilistic interpretation to the BBAs. Since in our setting $\left|\Theta \right|=2$, the BetP(H) map can be computed as: $\forall A\in \left\{H,\bar{H}\right\}$,

(10) $$BetP_{x}\left(A\right)=\frac{1}{1-m_{x}\left(Ø\right)}\left(m_{x}\left(A\right)+\frac{m_{x}\left(\Theta \right)}{2}\right).$$

This allows us to assign a $BetP_{x}\left(H\right)$ value to the resulting BBA associated to each pixel x that will be differently normalized on the basis of its conflict value, $m_{x}\left(Ø\right)$.

To illustrate the benefit of the explained BBA allocation for the CNN-ensemble, Table 2 proposes a toy example where MC-dropout is applied to sample the posterior distribution obtaining T = 4 realizations, for two different pixels $x_{1}$ and $x_{2}$. Then, discounting coefficients $\gamma_{x,t}$ are derived using Equation (7), setting α=0.5. After having performed the conjunctive combination among the discounted BBAs, $BetP_{x}\left(H\right)$ and $m_{x}\left(\Theta \right)$ are shown for the two pixels $x_{1}$ and $x_{2}$. The posterior distribution sampled for pixel $x_{1}$ presents similar values with respect to the one sampled for pixel $x_{2}$, so that all the realizations are close to the median value and thus we obtain high discounting coefficients that reflect in reliable BBAs that do not need to be much discounted. Conversely, $x_{2}$ presents a sampled distribution which is more spread out, so that more discounting (i.e., lower discounting coefficients) is applied. This fact reflects in higher value of ignorance for $x_{2}$, that may be interpreted as higher predictive uncertainty.

Now, instead of directly taking decision based on Equation (10), we keep $M_{CNN}$ aside for a later fusion with another ensemble of classifiers composing the proposed evidential MCS.

### 2.3. SVM-Ensemble

The second ensemble of the proposed evidential MCS is based on SVM classifiers and it is obtained through active learning, which we find complementary to deep networks being particularly adapted in situations where we have a specific problem and an extremely small training set. Through active learning indeed, we are able to select the most informative training samples in order to reduce at minimum the number of samples needed yet maximizing the overall detection performance.

The SVM-ensemble here employed has been previously proposed in [11], where an evidential Query-By-Committee active learning strategy is designed in order to exploit different detectors based on different descriptors (gradient, texture and orientation features) in order to train an ensemble of SVM-based classifiers with limited amount of data. In that work, the BFT is exploited both for the sources combination and for the new samples selection. The result of the source combination indeed is a BBA associated to every unlabeled sample, that intrinsically contains conflict and ignorance components.

We now briefly recall the BBA allocation proposed in [11] for the sake of clarity, as the BBA map $M_{SVM}$ resulting from the SVM-ensemble fusion will be the second input of the proposed evidential MCS.

#### 2.3.1. SVM-Ensemble BBA Allocation and Combination

In the context of SVM-based high density crowds pedestrian detection, imprecision can arise in two different and complementary ways: in the derivation of posterior probability values from SVM decision scores, and later, from the spatial layout of the detections in the output image space.

Two successive discounting steps are thus processed on the initial Bayesian BBAs derived from the learned sigmoids during Platt’s calibration [54]. Firstly, having learned the sigmoid $\sigma_{i}$ of classifier *i* by logistic regression, BBAs are defined to model the imprecision due to possible errors in the calibration, by applying erosion $E_{w}$ and dilation $\delta_{w}$ operators in the 2D space where SVM calibration scores are projected with respect to their label, with structuring element *w*. Then, the mass on Θ is increased by discounting the previous BBAs, by performing a morphological opening operation $\gamma_{a}$, this time in the image space, to take into account neighbor pixels information based on the assumption that they are likely to belong to the same class.

Specifically, with $s_{x}$ being the SVM score associated to pixel x:

$$\begin{array}{c} \left\{\begin{array}{ccc} \forall x\in P,{\tilde{m}}_{x,i}\left(H\right) & = & \left(E_{w}\circ \sigma_{i}\right)\left(s_{x}\right),\\ \forall x\in P,{\tilde{m}}_{x,i}\left(\bar{H}\right) & = & 1-\left(\delta_{w}\circ \sigma_{i}\right)\left(s_{x}\right),\\ \forall x\in P,{\tilde{m}}_{x,i}\left(\Theta \right) & = & 1-{\tilde{m}}_{x,i}\left(H\right)-{\tilde{m}}_{x,i}\left(\bar{H}\right). \end{array}\right. \end{array}$$

where P is the pixel domain, $\sigma_{i}$ is the learned sigmoid for classifier *i* and $\left(E_{w}\circ \sigma_{i}\right)$ and $\left(\delta_{w}\circ \sigma_{i}\right)$ its eroded and dilated results with a (flat) structuring element of width *w*, applied in the score space. Then, in the image space,

$$\begin{array}{c} \left\{\begin{array}{ccc} M_{i}\left(Ø\right) & = & {\left\{0\right\}}_{x\in P},\\ \forall A\in \left\{H,\bar{H}\right\},M_{i}\left(A\right) & = & \gamma_{a}\left(\tilde{M_{i}}\left(A\right)\right),\\ M_{i}\left(\Theta \right) & = & {\left\{1\right\}}_{x\in P}-M_{i}\left(H\right)-M_{i}\left(\bar{H}\right), \end{array}\right. \end{array}$$

where $M_{i}\left(A\right)$ is the layer image associated to hypothesis *A*, $\forall A\in 2^{\Theta }$, and $\gamma_{a}$ is the opening operator of parameter *a* applied in the image domain.

As in [11,55,56], a spatial Gaussian structuring element fitted in a window of radius *a* is used, to better take into account the spatial consistency.

The final result is thus a BBA map $M_{SVM}$, that can be used either for decision through Equation (10), or in conjunction with the previously defined $M_{CNN}$ to compose the evidential MCS.

### 2.4. Evidential MCS

Until now we have proposed two different ensemble-based methods based on two different classifiers, namely SVM and CNN. In order to obtain the final MCS, we intend to perform a fusion between the two ensembles. Note that this is not straightforward, since in presence of few, strong classifiers the fusion strategy must be particularly well-designed in order to exploit their respective strengths. Moreover, in this work a noticeable difficulty comes from the unbalanced performance between the two ensembles which makes most of fusion schemes not improving results derived from CNN approach alone.

**Figure 3:**
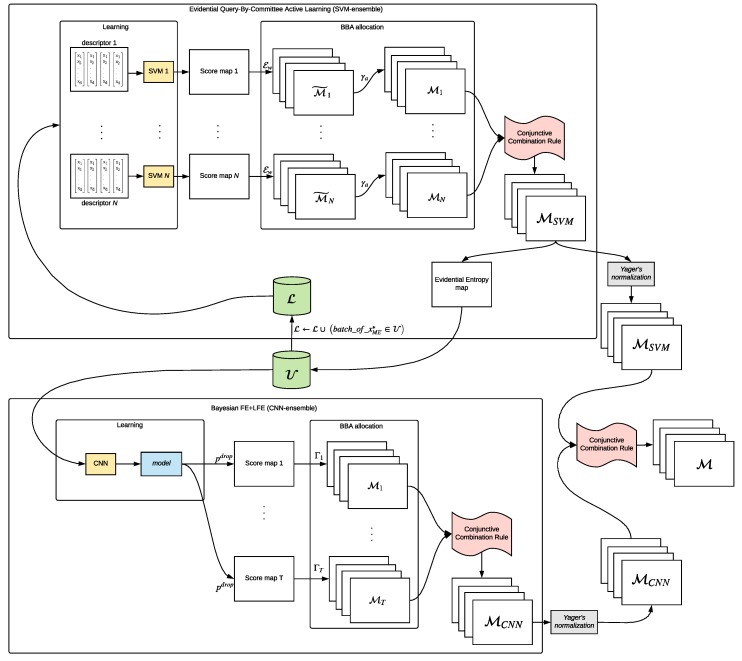
Proposed evidential Multiple Classifier System flowchart.

Figure 3 shows the overall flowchart of the final evidential MCS. Starting from the initial pool of samples U, we perform in a parallel way the SVM-based active learning procedure to select the most informative samples to be added to the set of labeled training samples L, while at the same time we train the FE+LFE network on the entire set U.

In order to build the SVM-ensemble, the evidential QBC active learning procedure consists of the following steps:

- Training the different SVM classifiers based on different features (e.g., HOG [13], LBP [57], DAISY [58] and GABOR [59] are employed in [11]);
- Performing BBA allocation for each pixel of each source, taking into account possible imprecision in the score calibration procedure and in the image space, and combining them through the conjunctive combination rule;
- Selecting the new samples to be added to the SVM training set L based on evidential entropy disagreement measures.

At the end of the evidential Query-by-Committee procedure, the result is a single four-layers BBA map ($M_{SVM}$) with a BBA associated to each pixel that intrinsically contains evidence of belonging to *H* and $\bar{H}$, i.e., $M_{SVM}\left(H\right)$ and $M_{SVM}\left(\bar{H}\right)$ respectively, as well as a component of ignorance ($M_{SVM}\left(\Theta \right)$) which is not solved through the combination and a component of conflict ($M_{SVM}\left(Ø\right)$) that arises through the combination itself.

Regarding the second component of the MCS, namely the CNN-ensemble, it also consists of several steps:

- Training the FE + LFE network (Section 2.2.3) based on the relatively small training dataset U;
- Applying MC-dropout procedure at inference time to obtain the *T* realizations, as explained in the previous Section 2.2.4;
- Performing BBA allocation for each realization to model the network’s predictive uncertainty about each pixel’s prediction, and combining them through the conjunctive combination rule (cf. Section 2.2.5).

The output of the proposed evidential CNN-ensemble is thus a single four-layers BBA map ($M_{CNN}$), where each pixel contains evidence of belonging to $Ø,H,\bar{H},\Theta$ respectively. Here we interpret the ignorance value related to each pixel as the model’s predictive uncertainty about it, being able thus to model the imprecision in addition to the uncertainty value provided by the network.

After having combined a relatively high number of sources through the conjunctive rule both to obtain $M_{SVM}$ and $M_{CNN}$, we note that they both contains non-negligible masses on the empty set representing the conflict while the mass on the Θ focal element naturally decreases thanks to the conjunctive combinations. This could lead to disproportionate values of conflict with respect to the masses on the other focal elements. To solve this issue, classically Dempster’s rule is adopted or a normalization of the BBAs is lately performed, but in this way the conflicting mass would be equally spread over the remaining hypothesis. Instead, as done in [53], we focus on the normalization included in Yager’s combination rule [60] that, in the absence of knowledge about the conflict origin, transfers it to the ignorance component.

Finally, the conjunctive combination rule is performed between the normalized $M_{SVM}$ and $M_{CNN}$, obtaining the final BBA map M which can be used either for decision, computing the associated BetP(H) map with Equation (10), and to obtain a measure of the imprecision about the final prediction, naturally given by M(Θ).

## 3. Results

### 3.1. Dataset

The proposed algorithm was tested on images acquired at one of the largest scale, high-density locations accessible for study, namely the holy Muslim pilgrimage taking place in Makkah, Saudi Arabia. The data was acquired at peak density during Hajj [61] in 2012. The camera used for recording is a robotic camera (AVT Guppy PRO) mounted statically in order to observe the high-density pilgrim crowd, and providing gray-level regular images (visible spectrum). The input data consists thus in a video sequence of the crowd (at a frame-rate of 8 Hz), but for the detection task we use individual images. For the training, calibration and evaluation of the head detectors, we use 35 images extracted at distant moments (in order to establish a full level of independence among the images used). Each image instance contains in the analyzed Region of Interest (corresponding roughly to the lower half of the scene) a high number of objects to detect (about 900–1000 heads) due to the high density.

The use of an ensemble, along with the discussed regularization techniques, allows us to apply a deep learning-based solution even in presence of a very small dataset. In particular, as training set we use the pool of data available for the active learning solution in [11] for choosing the new samples to add to the training set, noted U, i.e., the pool of unlabeled samples for the active learning which corresponds to image patches roughly including 2000 different heads. Note that in the traditional active learning an oracle is supposed to answer about the true label of a sample only when it has been chosen by the algorithm to be added to the set, so that U is indeed a pool of yet unlabeled samples. In our case, the pool is not unlabeled as we dispose of ground-truth maps, nevertheless for consistency we keep the notation “U”. Note also that the active learning solution do not use all the available data in U, but selects only 2000 positive or negative samples out of it (i.e., pixels belonging or not to a head). Nonetheless, we consider this a rather fair compromise since the training data available to the two methods is a-priori the same (while the active learning procedure chooses the most informative samples, the deep learning strategy uses all the available pixels of the training images in a fully convolutional architecture setting). Then, image patches containing roughly others 2000 heads (different from the training ones) are used for validation, and the rest of the dataset is used for testing.

**Figure 4:**
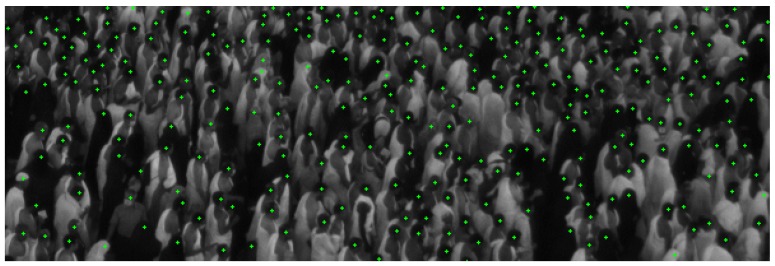
Patch with ground-truth dotted annotation.

The difficulty of the detection task results from multiple factors that we briefly introduce in the following paragraph. Figure 4 shows a patch from an image of the dataset, highlighting the difficulty of the problem since the heads are barely visible and many occlusions occur. We performed a dotted annotation in the head centers for the training images, such that the ground-truth so obtained can be used either as an *oracle* to assign the correct label to the samples selected for querying by AL (Active Learning), or in order to evaluate the loss for training the fully convolutional network. Even though in Makkah the crowd follows a general direction, there is a significant degree of head appearance variability due to gender, type of head cover, and most importantly, to the various degrees of occlusion coupled with the small size of the targets. For annotating a single image (clicking on the heads exhaustively), a human annotator requires typically half a day of work, and approximately 20% of the heads are so difficult to annotate that the human needs to look in the previous and the next frames in order to take a head/not head decision (something which our algorithm cannot do, as it performs the detection only in the current frame). As it is possible to see from the image, another problem in this type of scenes is the high data imbalance between positive and negative samples (i.e., pixels belonging or not to a head, respectively), stressing the importance of finding an effective strategy to select significant samples for SVM on one side, and exploiting at maximum the information of the few positive pixels with dilated convolutions for the fully convolutional deep architecture on the other side.

### 3.2. Evaluation Method

Regarding the evaluation procedure of the pedestrian detection map results, we choose to evaluate our method on the basis of two different measures, which do not depend on any threshold and at the same time are suited for imbalanced data, namely Area Under Precision-Recall Curve (AUPRC) and Precision-Recall Break Even Point (PRBEP). This latter in particular is a useful operative threshold value, corresponding to the threshold for which Precision is equal to Recall, the number of false positive detections (fp) is equal to the number of false negatives (fn) since $Precision=\frac{tp}{tp+fp}$ and $Recall=\frac{tp}{tp+fn}$ with tp the number of true positive detections. These two metrics are computed on the BetP(H) map, applying non maxima suppression (NMS) at every threshold to identify the targets, setting the radius of a head to r=3, with 2r+1 minimum distance between two maxima (head centers) in order to avoid overlapping detections. The value of the radius has been set empirically and depends on the dataset.

### 3.3. Training Setting

Fully convolutional networks usually cast segmentation as a dense *classification* problem, in the sense that each pixel is assigned to a given class (where the number of classes is discrete). For this reason, they commonly employ loss functions suited for this task, e.g., cross-entropy (weighted, in case of class imbalance). We are rather interested in performing *regression*, since after the cumulative Gaussian soft labeling the pixels of the ground-truth map (and thus our desired output) are not labeled with their class but rather with a real value resulting from the accumulation of head distributions. Since output values are not discrete (disregarding the unavoidable discretization of the Gaussian function over the pixel domain) and possibly not bounded, we choose to use a MSE loss, as an efficient pixelwise estimate of the distance between two 2D maps.

Note that the parameter η of Equation (1) is equivalent to weight the loss for the positive class for classification problem employing weighted cross-entropy loss. The parameter η is particularly important since higher its value, higher the impact of each single pixel belonging to a head in the loss function, and must be set taking into account the expected crowd density (lower the density higher its value, as per-pixel class imbalance would be more relevant). Considering that a head diameter spans between 8 and 12 pixels, pixel-level class imbalance issue is solved by setting η=150 in Equation (1) (value empirically obtained through validation).

The network is trained by using an Adam stochastic optimizer [62], with a learning rate of $7\times 10^{-3}$ for the proposed FE + LFE (exact values have been found through validation). The weights of the convolutional layers are initialized with the Kaiming He method [63], which has proven to be particularly adapted for deep networks relying on ReLU activation functions. Early stopping with a patience of 20 epochs is used in order to terminate the learning process when the networks stop improving on the validation set. This contributes also to mitigating the risk of overfitting.

Regarding the hyperparameters used to obtain the SVM-ensemble through active learning, we refer the reader to the previous work [11].

### 3.4. CNN-Ensemble Results

Before showing the results of the final evidential MCS, we firstly investigate the benefit of the proposed evidential CNN-ensemble over traditional methods, using the proposed FE + LFE network.

In order to obtain the CNN-ensemble, we applied MC-dropout method. Dropout is added in the central layers as in [32], i.e., before and after the bottleneck layer with dilation factor equal to 3 (see Table 1). The probability of dropout $p^{\mathrm{drop}}$ is set to 0.2 since the default value of 0.5 resulted to be detrimental for the final result. The number of realizations *T* is fixed to 10 through validation, as increasing it do not show any significant performance improvements.

**Figure 5:**
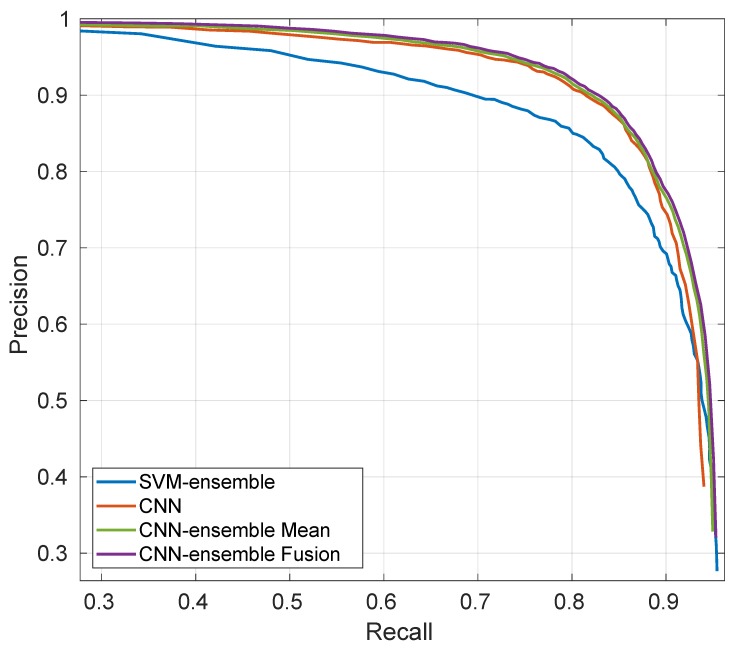
PR-curves of SVM-ensemble and deep learning solutions. All the classifiers disposed of the same amount of (limited) data for the training.

Figure 5 shows the PR-curves obtained with the active learning solution based on the use of a committee of SVMs [11] (denoted as “SVM-ensemble”) with the deep learning-based solutions obtained training the network on the same limited amount of data. Specifically, after training the FE + LFE network, “CNN” refers to the output map obtained with the traditional forward pass to perform inference. “CNN-ensemble Mean” and “CNN-ensemble Fusion” refer instead to the use of MC-dropout to obtain the ensemble, combining the members through the traditional average operator and with the proposed evidential approach respectively. Table 3 provides quantitative values for PRBEP and AUPRC with the same names notation.

As it is possible to see both from Figure 5 and from Table 3, deep learning-based solutions tends to outperform SVM-based one, most noticeably regarding the precision values. Nevertheless, SVM has been trained with a chosen fraction of the available samples pool U, with respect to deep learning-based methods that are able to exploit all the available data.

The use of the CNN-ensemble to perform inference rather than the usual forward pass is beneficial especially in increasing the recall values, meaning that the ensemble is able to retrieve more heads. Note that there is not a great difference between the mean output map $M_{\mu }$ (CNN-ensemble Mean) and the BetP(H) map after having performed the fusion of the *T* realizations (CNN-ensemble Fusion), although this latter is slightly better. However, having defined a BBA allocation for each realization allows us to have a final BBA map that can be easily combined together with the BBA map given by the SVM-ensemble.

**Figure 6:**
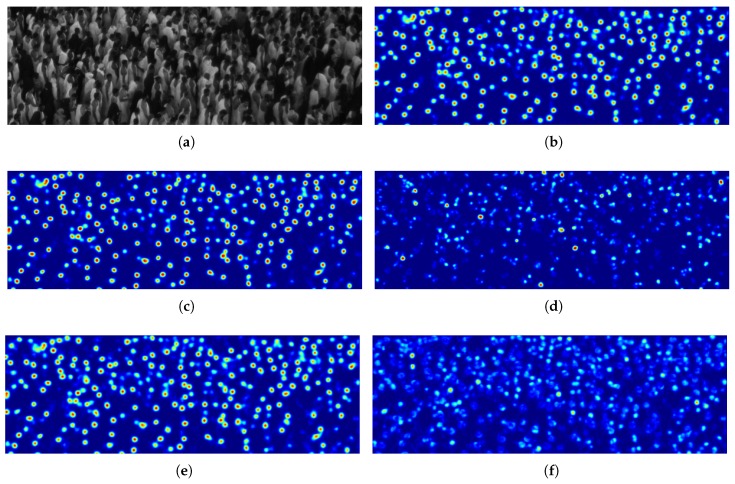
Output maps on a testing image patch with the deep learning solutions trained on the same amount of limited data, as well as model’s predictive uncertainty outputs through traditional standard deviation and proposed evidential ignorance. (**a**) Image patch; (**b**) Output map of FE + LFE; (**c**) Output map of CNN-ensemble Fusion, i.e., BetP(H); (**d**) Ignorance map of CNN-ensemble Fusion, i.e., M(Θ); (**e**) Output map of CNN-ensemble Mean, i.e., $M_{\mu }$; (**f**) Standard deviation map of CNN-ensemble, i.e., $M_{\sigma }$.

Figure 6 shows the final output maps for a given image patch, considering the traditional forward pass for inference in Figure 6b with respect to the CNN-ensemble based output maps in Figure 6c,e, respectively obtained through the proposed BBA allocation and evidential fusion, and through the classical average of the *T* realizations. Figure 6d,f instead, represent the ignorance map after the evidential conjunctive combination and the classical standard deviation map respectively, which can be interpreted as a measure of predictive uncertainty. The predictive uncertainty map obtained with the proposed evidential method is clearly more precise in the localization of areas where the model is uncertain, while the standard deviation map although being useful is less localized and noisier.

**Figure 7:**
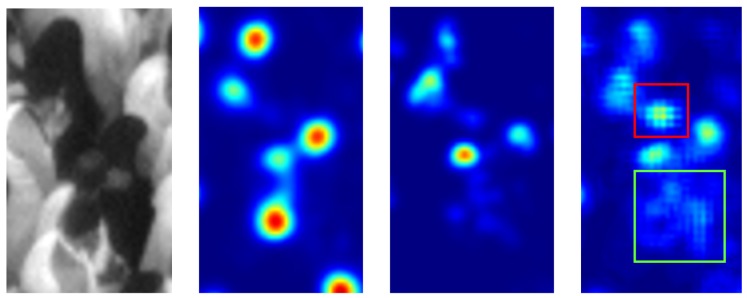
From left to right: (**a**) Example of an image patch (with increased contrast for clarity) with (**b**) associated output map of CNN-ensemble Fusion, i.e., BetP(H), (**c**) ignorance map (proposed) and (**d**) traditional standard deviation map respectively. Failure cases of standard deviation map are highlighted in red and green boxes.

This behaviour is even better highlighted in Figure 7, where for a given little image patch we see the associated output map of CNN-ensemble Fusion, i.e., BetP(H), the ignorance map obtained as M(Θ) and the traditional standard deviation map. The ignorance map is far less noisier than the standard deviation map, as highlighted especially in the area of the green box. Moreover, the standard deviation map provides misleading high values in some areas (e.g., the highlighted red box), where the network ensemble correctly predict no heads (and indeed the associated ignorance is correctly low). The areas of the ignorance map with high values on the contrary are more localized, and generally correspond to difficult cases such as occluded and low-contrasted heads.

### 3.5. Evidential MCS Results

**Figure 8:**
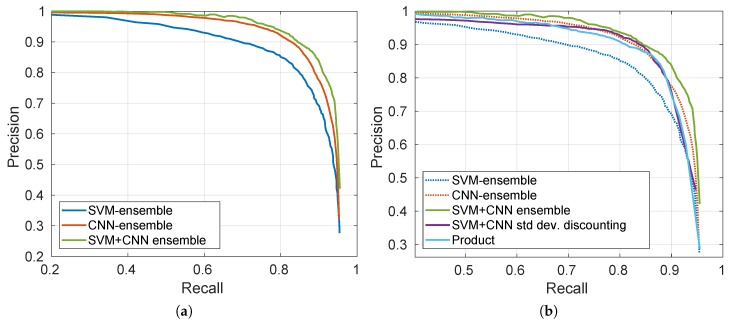
(**a**) PR-curves of SVM-ensemble and CNN-ensemble, along with their combination SVM+CNN ensemble; (**b**) Comparison in terms of PR-curves of the proposed SVM+CNN ensemble with respect to product of BetP(H) maps given by the two ensembles, and a fusion between the SVM-ensemble BetP(H) map with the result of a simple discounting performed on the mean map $M_{\mu }$ based on the standard deviation values in $M_{\sigma }$.

To illustrate the benefit of the final evidential MCS, Figure 8a shows the Precision Recall (PR) curve of the proposed approach described with the flowchart reported in Figure 3, where the SVM-ensemble and CNN-ensemble BBA output maps are combined together after Yager’s normalization. PR-curves of SVM-ensemble and CNN-ensemble alone are reported as well, to show the improvement obtained thanks to their fusion.

Figure 8b shows the comparison of the proposed approach with respect to two other strategies, namely the fusion between SVM-ensemble and the result of a simple discounting performed on the mean map $M_{\mu }$ based on the standard deviation values in $M_{\sigma }$, and the product of BetP(H) maps (interpreted as probability maps) given by the two ensembles. The two initial sources SVM-ensemble and CNN-ensemble are reported as well with dotted lines. Values of PRBEP and AUPRC for the considered approaches are then detailed in Table 4.

Both from the PR-curves and from the values reported in the table, we can see that the evidential fusion of the two ensembles preceded by Yager’s normalization resulted to be the best approach. Conversely, both the product of probabilities and the simpler discounting method fail to exploit all the available information so that the final result do not improve on CNN-ensemble or rather worsen it. This is due to the fact that, being already a map of BBAs obtained after the fusion of the *T* realizations, CNN-ensemble’s BetP(H) map is more informative than the mean map on which we apply a hand-crafted discounting (even though tailored with respect to standard deviation).

This proves that, in presence of few labeled data, the joint use of two classifiers (in our case SVM and CNN) is able to reach competitive performance.

**Figure 9:**
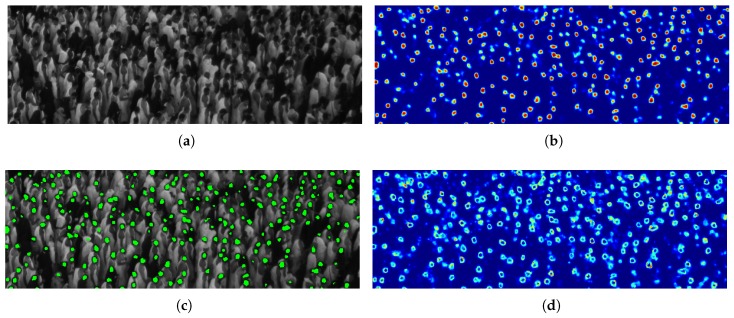
Visual results obtained testing the proposed evidential MCS on an image patch, in terms of BetP(H) output map, detection map at the threshold corresponding to the PRBEP, and the final ignorance map of the system. Output maps on a testing patch image with the deep learning solutions trained on the same amount of limited data, as well as model’s predictive uncertainty outputs through traditional standard deviation and proposed evidential ignorance. (**a**) Image patch; (**b**) Output map of SVM+CNN ensemble, i.e., BetP(H); (**c**) Detections at PRBEP threshold; (**d**) Ignorance map of SVM+CNN ensemble, i.e., M(Θ).

Figure 9 provides visual results obtained testing the proposed evidential MCS on a given image patch, in terms of BetP(H) output map, detection map at the threshold corresponding to the PRBEP, and the final ignorance map of the system. The obtained BetP(H) map presents well-localized and well-shaped detections. Regarding the ignorance map, which we interpret as the global system’s predictive uncertainty, we notice that it presents higher values in the surrounding of the heads. This is due to the fact that we applied Yager’s normalization before the combination of the two ensembles based on the different classifiers, reversing the conflict mass (which is higher at the border of the heads) on the compound set. Thus, a part of the ignorance is not solved with the final combination resulting in the obtained map. Nevertheless, disregarding from the high values on the head’s borders, the map is interesting in that it highlights the regions where none of the classifiers (nor the SVM nor the deep learning-based one) were able to give a committed answer about the predicted pixel’s value.

## 4. Conclusions

In this work, we proposed an evidential Multiple Classifier System which, based on the joint use of two heterogeneous classifier ensembles, is able to reach a higher level of performance that would otherwise be accessible only by using larger amounts of annotated training data. On the first hand, our approach underlines the importance of modeling uncertainty in fusion rules for decision systems for extracting additional information from prior knowledge and training data. Secondly, the pedestrian detection application we considered shows the practical interest of such methods for deploying classifiers with a lower burden in terms of required labeling, which is often a costly and time-consuming process. Ultimately however, the main aim of our work is not necessarily to provide a better classifier, but mostly to provide a robust approach that works in presence of limited training datasets and which is also able to give insights related to the interpretability of deep learning-based methods, addressing the limitations raised by standard deep learning architectures, which tend to perform as “black boxes”.

In future studies, we intend to tackle cross camera detection, a task which is far from trivial at high densities due to scene scale and illumination changes and to significant variations in occluded pedestrian appearance. At the same time, an easy fine-tuning process among different views is vital from a practical standpoint. Since our method requires less training data in order to reach a good performance, we intend to investigate whether the proposed strategy may be extended as well during a retraining step, in order to minimize thus the amount of costly annotations required in order to deploy a detection system in additional views.

## Figures and Tables

**Table 1:** Detailed architecture of the proposed network inspired by [41], where *F* is the number of filters and *D* is the dilation factor to perform dilated convolutions. It is possible to notice the symmetric structure of the dilations whose factor increases in the Front End (FE) module, allowing us to increase the receptive field, and decreases in the Local Feature Extraction (LFE) module, aggregating local features to obtain spatial consistency in the output map. Note that each convolutional layer is followed by batch normalization (except the last layer) and ReLU activation function.

	Layers
FE	Conv 3×3, F=16, D=1
	Conv 3×3, F=32, D=1
	Conv 3×3, F=32, D=2
	Conv 3×3, F=64, D=2
	Conv 3×3, F=64, D=3
LFE	Conv 3×3, F=64, D=2
	Conv 3×3, F=64, D=2
	Conv 3×3, F=64, D=1
	Conv 3×3, F=64, D=1
	Conv 1×1, F=1, D=1

**Table 2:** Example of different values obtained sampling the posterior distribution with MC-dropout technique with T = 4, for two different pixels $x_{1}$ and $x_{2}$, along with the corresponding discounting coefficient $\gamma_{x,t}$ obtained with Equation (7) setting α=0.5. After having performed the conjunctive combination among the discounted BBAs, $BetP_{x}\left(H\right)$ and $m_{x}\left(\Theta \right)$ results are shown for the two pixels $x_{1}$ and $x_{2}$.

	t=1	t=2	t=3	t=4	Median	$\gamma_{x,1}$	$\gamma_{x,2}$	$\gamma_{x,3}$	$\gamma_{x,4}$	${\mathrm{BetP}}_{x}\left(H\right)$	$m_{x}\left(\Theta \right)$
$x_{1}$	0.8	0.8	0.82	0.82	0.81	0.99	0.99	0.99	0.99	0.87	0.06
$x_{2}$	0.01	0.99	0.27	0.73	0.5	0.51	0.51	0.77	0.77	0.5	0.2

**Table 3:** Precision-Recall Break Even Point and Area Under Precision-Recall Curve with the different architectures trained on the same limited amount of data.

	SVM-Ensemble	CNN	CNN-Ensemble Mean	CNN-Ensemble Fusion
PRBEP	0.81	0.85	0.85	0.86
AUPRC	0.86	0.89	0.90	0.90

**Table 4:** Precision-Recall Break Even Point and Area Under Precision-Recall Curve of the BetP(H) result with the proposed MCS composed by SVM+CNN ensemble, as well as a comparison with respect to product of BetP(H) maps given by the two ensembles, and a fusion between the SVM-ensemble BetP(H) map with the result of a simple discounting performed on the mean map $M_{\mu }$ based on the standard deviation values in $M_{\sigma }$. SVM-ensemble and CNN-ensemble performances are reported as reference.

	SVM-Ens.	CNN-Ens.	SVM+CNN Ens.	SVM+CNN Std Dev. Disc.	Product
PRBEP	0.81	0.86	0.87	0.86	0.86
AUPRC	0.86	0.90	0.92	0.89	0.90

## Abbreviations

The following abbreviations are used in this manuscript:

CNN	Convolutional Neural Network
BNN	Bayesian Neural Network
BFT	Belief Function Theory
MCS	Multiple Classifier System
AL	Active Learning
HOG	Histogram of Oriented Gradients
RPN	Region Proposal Network
SVM	Support Vector Machine
BBA	Basic Belief Assignment
NN	Neural Network
SRT	Stochastic Regularization Technique
MC	Monte Carlo
FE	Front End
LFE	Local Feature Extraction
MAD	Median Absolute Deviation
AUPRC	Area Under Precision-Recall Curve
PRBEP	Precision-Recall Break Even Point
NMS	Non Maxima Suppression
PR-curve	Precision Recall curve
